# Pelvic Discontinuity Caused by Acetabular Overreaming during Primary Total Hip Arthroplasty

**DOI:** 10.1155/2011/939202

**Published:** 2011-12-21

**Authors:** Iori Takigami, Yoshiki Ito, Takashi Mizoguchi, Katsuji Shimizu

**Affiliations:** ^1^Department of Orthopaedic Surgery, Gifu University School of Medicine, 1-1 Yanagido Gifu 501-1194, Japan; ^2^Department of Orthopaedic Surgery, Gifu Red Cross Hospital, Gifu 502-8511, Japan

## Abstract

Intraoperative acetabular fracture is a rare complication of primary total hip arthroplasty (THA), typically occurring during impaction of the cementless acetabular component. Here we report an unusual case of pelvic discontinuity caused by overreaming of the acetabulum during primary THA. Restoration of posterior columnar continuity was achieved with an autologous fibular graft and a reconstruction plate. Wall defects and cavitary defects were reconstructed with metal mesh and femoral head allograft, followed by placement and fixation of a Kerboull-type acetabular reinforcement device. Previous reports of acetabular fracture during THA have indicated that it has a relatively good prognosis without extensive treatment. However, to our knowledge, there has been no report of pelvic discontinuity necessitating acetabular reconstruction surgery as an intraoperative complication of primary THA.

## 1. Introduction

Intraoperative occurrence of an acetabular fracture is a rare complication of primary total hip arthroplasty (THA). Previous studies have demonstrated that underreaming of the acetabulum and insertion of an oversized acetabular component without cement may predispose to intraoperative acetabular fracture [1–3]. Good fracture healing and uneventful osseous ingrowth can be obtained with appropriate treatment such as weight-bearing restriction, change of the acetabular component, or addition of supplementary fixation screws, even if a fracture does occur [3, 4]. Herein, we present the first reported case of pelvic discontinuity due to overreaming of the acetabulum during primary THA, which was treated successfully by acetabular reconstruction surgery.

## 2. Case Presentation

The patient, a 77-year-old woman, had presented at a local hospital with severe pain and limited range of motion in her right hip. Plain radiography showed osteoarthritis in the right hip joint. Cementless total hip arthroplasty using a posterior approach was planned at that hospital. Intraoperatively, the surgeon overreamed the acetabulum because of insufficient visualization and recognized that reconstruction surgery would be required because of large acetabular defects. Bipolar hemiarthroplasty was performed with the aim of maintaining soft tissue tension, and thereafter the patient was referred to our hospital.

**Figure 1:**
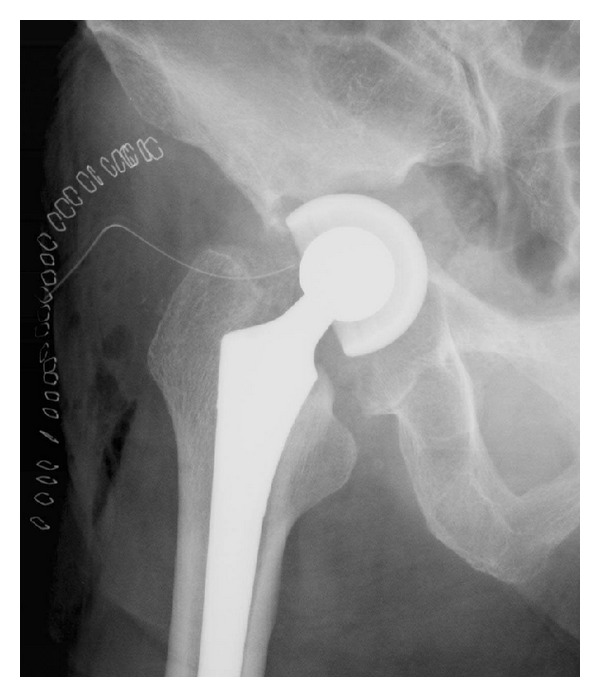
Anteroposterior radiograph showing severe acetabular bone loss.

**Figure 2:**
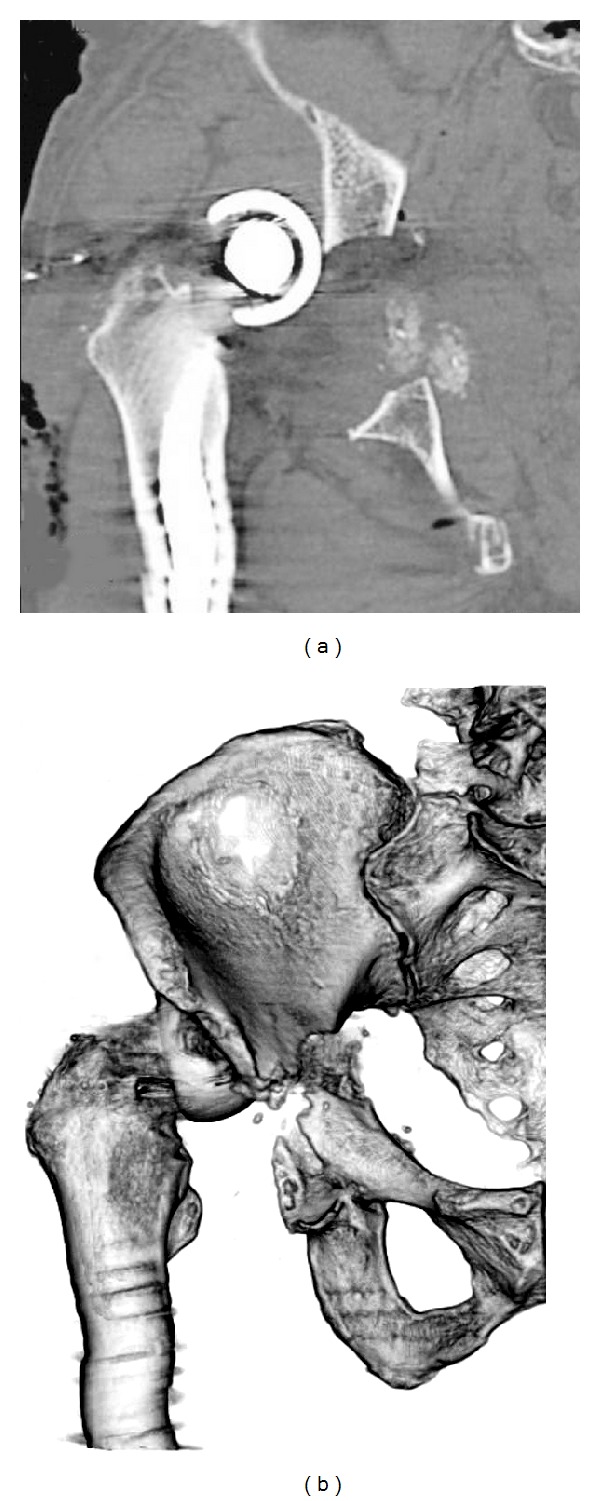
MDCT images showing pelvic discontinuity. (a) Coronal multiplanar reconstruction computed tomography. (b) 3D image in the anteroposterior view.

**Figure 3:**
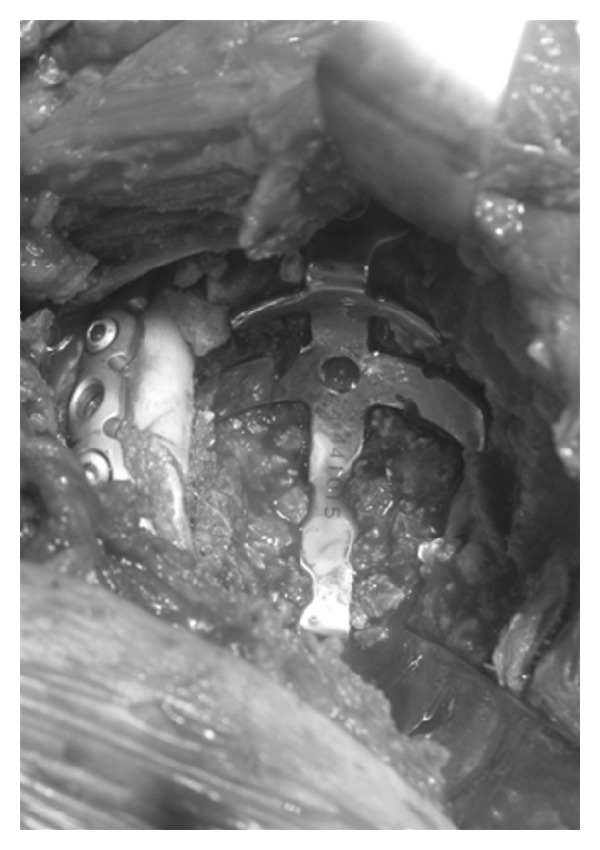
Intraoperative photograph of acetabular reconstruction.

**Figure 4:**
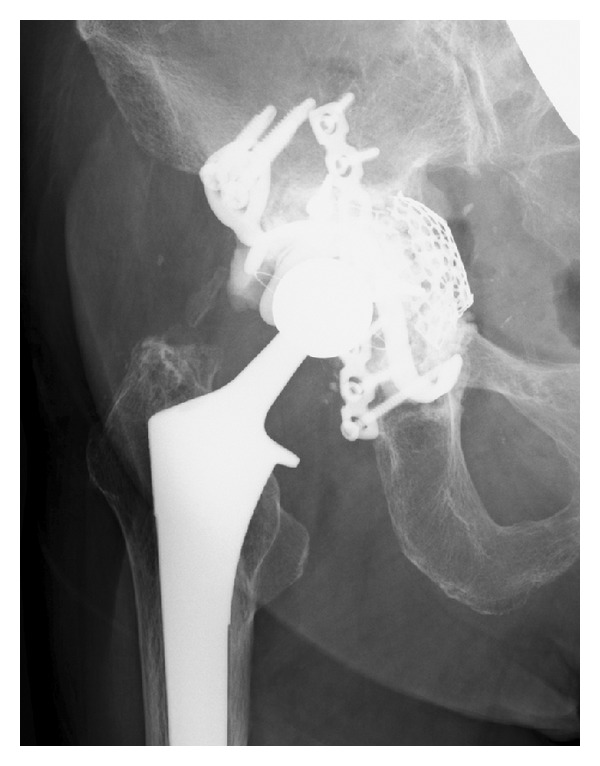
Latest radiograph of the hip taken 3 years after the operation. There is no migration or loosening of the prosthesis.

 X-ray imaging showed massive acetabular bone loss in the right hip joint (Figure 1). Multidetector-row computed tomography (MDCT) revealed pelvic discontinuity with an extensive bone defect (Figures 2(a) and 2(b)). Acetabular reconstruction surgery was performed using a posterior approach. Restoration of posterior columnar continuity was achieved with an autologous fibular graft and application of a posterior reconstruction plate to the ilium and ischium. The wall defects and cavitary defects were reconstructed with metal mesh and femoral head allograft, and then a Kerboull-type acetabular reinforcement device (KT plate) [5, 6] was placed and fixed with three screws (Figure 3). After these reconstructions, the polyethylene cup was cemented. Partial weight bearing was started 8 weeks after surgery, and full weight bearing was allowed after 16 weeks. The Japanese Orthopaedic Association hip score (range, 0–100 points) [7] improved from 40 points before the primary operation to 69 points 3 years after surgery. The latest radiograph showed complete bone union and no loosening of the components (Figure 4), and the patient is able to walk with single T-cane.

## 3. Discussion

Recently, cementless acetabular components have been widely used for THA. Porous coating can be used for biologically fixing the component to the host bone. It has been clearly shown that the amount of bone ingrowth that occurs into cementless implants is related to the initial stability of the component and the degree of metal apposition to cancellous bone [8, 9]. Polyethylene wear and osteolysis in THA have also been well documented. Multiple factors are responsible for the generation of wear and osteolysis. Several studies have indicated that increased linear polyethylene wear is correlated with a larger head and reduced polyethylene thickness [10–12]. To address these concerns, a larger acetabular component that enables the use of thicker polyethylene liner compared with a cemented cup is preferable to obtain better bone containment and initial stability.

Intraoperative fracture of the acetabulum is a very rare complication of THA. Previous studies have shown that it can typically occur during insertion of an oversized cementless acetabular component [2, 3]. Haidukewych et al. [4] reported a significant increase in the rate of intraoperative fracture with the use of designs involving a peripheral elliptical flare that was inserted into a bed that had been prepared with a hemispherical reamer. However, previous reports of acetabular fracture during THA have indicated that it has a relatively good prognosis without extensive treatment [3, 4].

In the present case, inappropriate acetabular reaming caused pelvic discontinuity with a massive bone defect. Desai and Ries reported two cases of early postoperative acetabular discontinuity after THA [13]. However, to our knowledge, pelvic discontinuity necessitating acetabular reconstruction surgery as an intraoperative complication of primary THA has not been reported previously. Treatment of pelvic discontinuity is difficult because there is a need to address both a pelvic fracture and hip revision arthroplasty simultaneously in the setting of massive bone loss. However, the best approach for the management of pelvic discontinuity is still unclear. For successful treatment, there is a need to achieve initial stability of the socket, to establish conditions for long-term socket stability, to stabilize any pelvic dissociation, and to restore the bone stock. In the present case, stabilization of the pelvic dissociation was achieved using an autologous fibular graft and a posterior pelvic reconstruction plate. The acetabular bone defect was reconstructed with metal mesh and femoral head allograft supported by a Kerboull-type acetabular reinforcement device. This device has been used for acetabular reconstruction in revision THA with excellent results [5, 14]. It protects grafts from overstress, working as a guide for hip reconstruction with an appropriate center of rotation, and stabilizes the reconstructed acetabulum [5, 14]. Moreover, Kerboull et al. [14] pointed out that this device provides enough rigidity to stabilize any pelvic discontinuity.

In this case, using acetabular reconstruction, we were able to treat successfully the pelvic discontinuity that had been caused by overreaming during primary THA. Although this type of situation is rather uncommon, care should be taken to avoid accidental overreaming of the acetabulum and thus creating a severe bone defect, especially in minimally invasive THA when it is difficult to totally visualize the surgical site. Acetabular bone deficiency caused by an inappropriate operative procedure is rare, but precise reconstruction is vital if ever it occurs, in order to ensure restoration of bone stock and long-term stability.
